# Relationship between CRP gene polymorphisms and ischemic stroke risk: A systematic review and meta-analysis

**DOI:** 10.1515/biol-2022-0505

**Published:** 2022-11-16

**Authors:** Zhizhi Chen, Feifei Jiang, Ming Yang, Jie Yang

**Affiliations:** Department of Neurology, The Quzhou Affiliated Hospital of Wenzhou Medical University, Quzhou People’s Hospital, Quzhou 324000, Zhejiang, China; Department of Rehabilitation Medicine, The Quzhou Affiliated Hospital of Wenzhou Medical University, Quzhou People’s Hospital, 100 Minjiang Road, Quzhou 324000, Zhejiang, China

**Keywords:** ischemic stroke, C-reactive protein, polymorphism, meta-analysis

## Abstract

Ischemic stroke (IS), usually caused due to an abrupt blockage of an artery, is the leading cause of disability and the second leading cause of death worldwide. The association of the C-reactive protein (CRP) gene (s3093059 T/C and rs1205 C/T) polymorphisms and IS susceptibility has been widely studied, but the results remain inconsistent. Our study aimed to assess the association between CRP gene (s3093059 T/C and rs1205 C/T) polymorphisms and IS risk. PubMed, Embase, Cochrane Library, Web of Science, China National Knowledge Infrastructure, and WanFang databases were searched up to April 2022 to identify eligible studies. The Newcastle-Ottawa scale (NOS) score was calculated to assess study quality. The odd ratios (ORs) with a 95% confidence interval (CI) were calculated to assess the association between CRP gene (rs3093059 T/C and rs1205 C/T) polymorphisms and IS risk. Eighteen case–control studies with 6339 cases and 29580 controls were identified. We found that CRP (s3093059 T/C and rs1205 C/T) polymorphism was not significantly associated with the risk of IS in any genetic model (recessive model: OR 1.00, 95% CI 0.79–1.26; OR 1.06, 95% CI 0.90–1.25). When stratified analysis by country, genotype method, source of controls, and NOS score, still no statistically significant association was found. Our study indicated that the CRP (rs3093059 T/C and rs1205 C/T) polymorphisms were not associated with the susceptibility to IS.

## 1 Introduction

Ischemic stroke (IS) is the more common type and is regarded as the leading cause of death, physical disability, and cognitive decline worldwide [1]. With the global population aged 65 and over growing faster than all other age groups, the incidence of stroke is also increasing. Accordingly, early accurate identification of modifiable risk factors and management of the people potentially at high risk of stroke are of great significance. There is strong evidence of a connection between the chronically activated and sustained inflammatory states and a variety of diseases including cancer [2], neurodegenerative disease [3], and cardiovascular and cerebrovascular disease [4]. The inflammatory processes have been observed in atherosclerotic initiation, plaque rupture, platelet activation, and coagulation system activation, which all contribute to the occurrence of IS [5]. Thus, inflammatory factor C-reactive protein (CRP), as one of the underlying circulating inflammatory markers, was identified to be a reactant in an acute phase of IS. In addition, elevated CRP levels are also generally associated with poor outcomes in acute IS patients [6]. However, the controversies have shown differences with respect to the risk prediction of IS because of genetic factors that influence CRP levels [7]. For example, some studies suggested that there was a positive relationship between elevated CRP and atherosclerosis as a precursor to IS [8]. Also, some showed that high-sensitivity CRP was not associated with IS and atherosclerotic changes [9,10]. Therefore, we speculated that the concentration of CRP in plasma depends on the CRP gene polymorphism. So far, approximately 30 single nucleotide polymorphisms (SNPs) of the CRP gene have been confirmed [11]. Their gene variability could be considered a predictive genetic marker for IS, so investigating the relationship between crucial binding sites SNPs and IS susceptibility may have diagnostic and prognostic implications.

Understanding the relationship between gene polymorphism and disease may help inform the design of pharmacotherapies by using multiple silico techniques [12,13,14]. A number of studies have been conducted to investigate the potential associations between common polymorphisms (rs2794521 (717G>A), rs3091244 (286CT>A), rs1800947 (1,059G>C), rs1130864 (1,444C>T)) in CRP gene and IS risk [15,16]. However, there is still a lack of summary conclusions about the association of CRP rs3093059 (757T>C) and rs1205 C/T polymorphism (2147C>T) with IS risk, though these SNPs polymorphism has also been proposed as possible biomarkers to predict IS risk in some researches. Therefore, we aimed to evaluate whether these two sites’ polymorphisms are associated with the risk of IS through a meta-analysis using all available data.

## 2 Materials and methods

This meta-analysis was conducted based on the Preferred Reporting Items for Systematic Reviews and Meta-Analyses (PRISMA) statement [17].

### 2.1 Literature search

A systematic literature search was conducted by using the combination of the following terms: “CRP,”, “CRP” “rs3093059,” “rs1205,” “polymorphism,” “variant,” and “IS” based on PubMed, Embase, Cochrane Library, Web of Science, China National Knowledge Infrastructure, and WanFang databases before April 2022. There were no language restrictions during the literature search. Additionally, the references of relevant articles were manually searched for potential studies.

### 2.2 Inclusion and Exclusion Criteria

The criteria for including studies in this meta-analysis were as follows: (1) the design of the study was case–control or cohort studies; (2) studies assessed the association between CRE gene polymorphism (rs3093059 or rs1205) and IS risk; (3) studies provided available genotype distribution in cases and controls; (4) the genotype distribution of control group conformed to Hardy–Weinberg equilibrium (HWE). The exclusion criteria were (1) studies that reported incomplete data or without data in cases and controls group; (2) duplicate data; and (3) review, case reports, or animal experiments.

### 2.3 Data extraction and quality assessment

Two authors independently extracted the following information from included studies: first author’s name, year of publication, country, ethnicity, genotype methods, genotype counts in cases groups and control groups, HWE results for control groups, and Newcastle-Ottawa scale (NOS) assessment. The NOS was calculated for the quality assessment of included studies. Discrepancies were resolved by consensus.

### 2.4 Statistical analyses

Meta-analyses were performed using the STATA version 12.0 (Stata Corporation, College Station, TX, USA), with a value of *p* < 0.05 which was considered statistically significant. To estimate a summary effect size for IS risk, the odds ratios (ORs) with 95% confidence intervals (CIs) were calculated by using the command “*metan”* based on five genetic models: allelic model, heterozygous model, homozygous model, dominant model, and recessive model. The significance of the pooled OR was determined by *Z*-test. Between-study heterogeneities were evaluated with *I* ^2^ statistic and Cochran’s Chi-square-based *Q* test. A fixed-effect model (Mantel–Haenszel method) was used when *I* ^2^ was ≤ 50%. Otherwise, analyses would be performed with random-effect models (Mantel–Haenszel method). HWE was tested by Chi-square test in controls. Sensitivity analysis was used to verify the stabilities of synthetic results. Publication bias was assessed using Begg’s funnel plots and Egger’s regression by “*metafunnel*” and “*metabias*” commands. We also conducted subgroup analyses by country, genotype method, source of controls, and NOS score. Trim-and-fill method was performed to adjust OR value when publication bias was found.

## 3 Results

### 3.1 Characteristics of included studies

**Figure 1:**
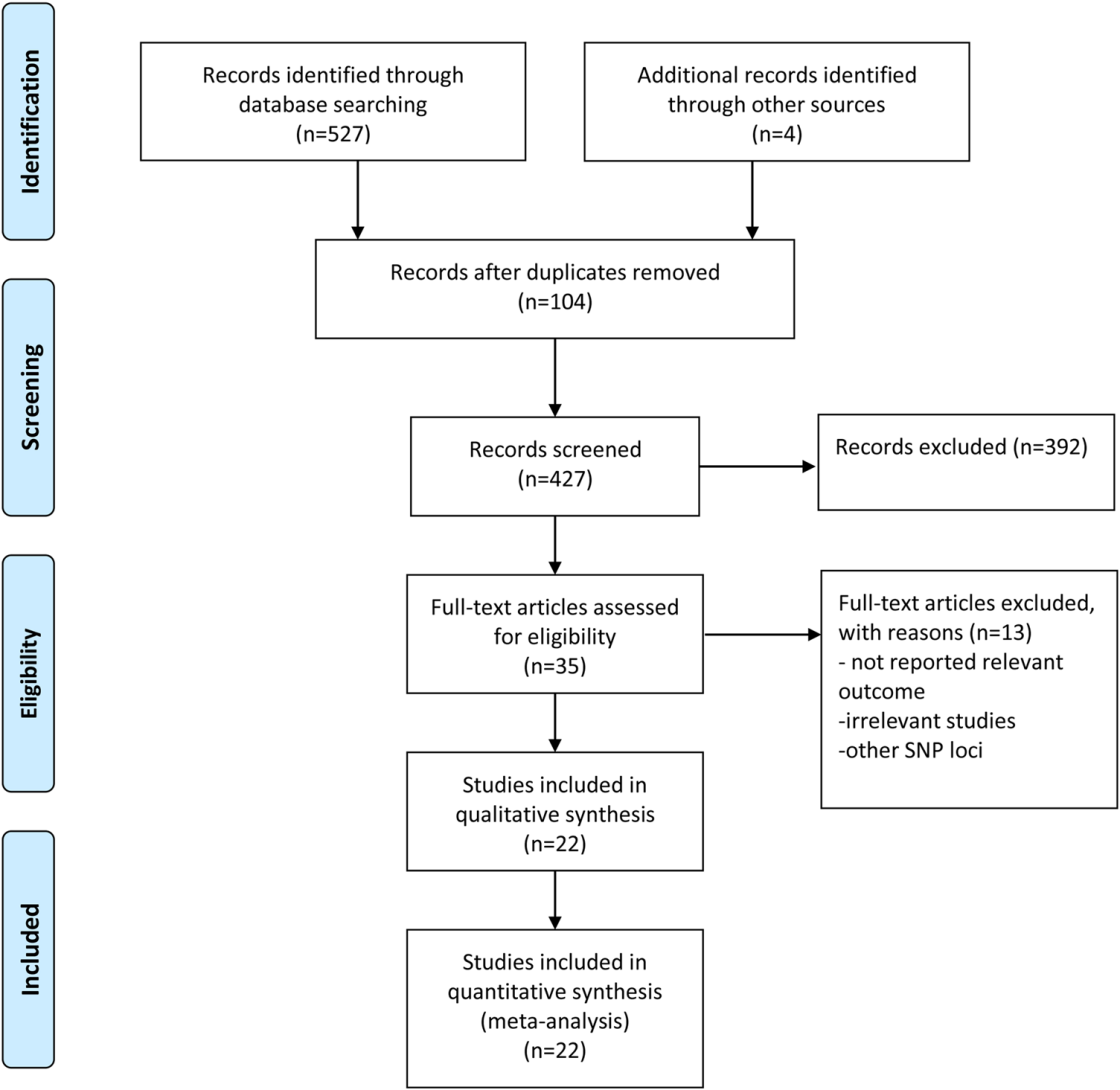
Flowchart of study selection.

By retrieving relevant databases, 531 possible related articles were initially identified. 104 were excluded due to duplication, and then 398 articles were excluded through screening title and abstract. Finally, 18 articles [11,18,19,20,21,22,23,24,25,26,27,28,29,30,31,32,33,34] were included in this meta-analysis (Figure 1). As shown in Table 1, nine studies focused on rs3093059 T/C polymorphism (including 3,109 patients and 4,939 controls), and 12 studies focused on rs1205 C/T polymorphism (including 4346 patients and 25870 controls). These studies were published from 2006 to 2016, and NOS scores ranged from 6 to 8 points. All the control populations were consistent with HWE. All the studies were conducted on Asians. The studies were carried out in China and Japan.

**Table 1:** Characteristics of the investigated studies of the association between the CRP (rs3093059, rs1205) polymorphisms and IS risk

First author	Year	Country	Ethnicity	Genotype method	Source of controls	Case	Control	Case			Control			HWE	NOS
Rs3093059								TT	TC	CC	TT	TC	CC		
Jiang et al.	2014	China	Asian	PCR-RFLP	HB	548	993	387	148	13	648	313	32	0.435	7
Chen et al.	2015	China	Asian	PCR-RFLP	HB	159	175	108	48	3	115	56	4	0.349	6
Zhao et al.	2018	China	Asian	Mass ARRAY	HB	373	613	263	96	14	431	169	13	0.449	7
Li et al.	2013	China	Asian	PCR-RFLP	HB	129	192	54	51	24	99	70	23	0.061	6
Jiang et al.	2012	China	Asian	PCR-RFLP	PB	510	994	362	135	13	649	314	31	0.346	7
Du et al.	2015	China	Asian	PCR-RFLP	HB	158	290	101	52	5	200	86	4	0.118	6
Wu et al.	2017	China	Asian	TaqMan	PB	580	582	382	172	26	301	238	43	0.666	7
Shen et al.	2009	China	Asian	PCR-RFLP	PB	552	994	386	148	18	649	314	31	0.346	6
Huang et al.	2016	China	Asian	TaqMan	HB	100	106	52	39	9	67	34	5	0.798	8
Rs1205								CC	CT	TT	CC	CT	CC		
Wu et al.	2010	China	Asian	PCR-RFLP	HB	150	125	74	67	9	57	55	13	0.960	7
Liu et al.	2015	China	Asian	PCR-RFLP	HB	60	12	16	29	15	17	31	17	0.753	6
Xu et al.	2015	China	Asian	PCR-RFLP	HB	113	113	20	52	41	19	58	36	0.593	7
Wu et al.	2017	China	Asian	TaqMan	PB	580	582	30	172	378	53	222	307	0.165	7
Huang et al.	2016	China	Asian	TaqMan	HB	100	106	49	38	13	47	40	19	0.052	8
Luo et al.	2015	China	Asian	PCR-RFLP	HB	113	113	20	52	41	19	58	36	0.593	6
Deng et al.	2012	China	Asian	PCR-RFLP	HB	105	121	20	47	38	20	62	39	0.577	7
Yu et al.	2012	China	Asian	PCR-RFLP	HB	1,572	1,485	548	729	295	512	715	258	0.757	6
Zhao et al.	2018	China	Asian	Mass ARRAY	HB	376	613	61	187	128	104	285	224	0.413	7
Wang et al.	2009	China	Asian	TaqMan	HB	564	564	110	282	172	94	297	173	0.078	6
Morita et al.	2006	Japan	Asian	TaqMan	PB	152	304	72	68	12	137	125	42	0.122	7
Miller et al.	2005	Japan	Asian	TaqMan	PB	461	21,732	212	191	58	9,700	9,580	2,452	0.238	8

HWE, Hardy–Weinberg equilibrium; HB, hospital-based source of control; PB, population-based source of control; PCR-RFLP, polymerase chain reaction-restriction fragment length polymorphism; NOS, Newcastle-Ottawa scale.

### 3.2 CRP rs3093059 T/C polymorphism and IS risk

**Figure 2:**
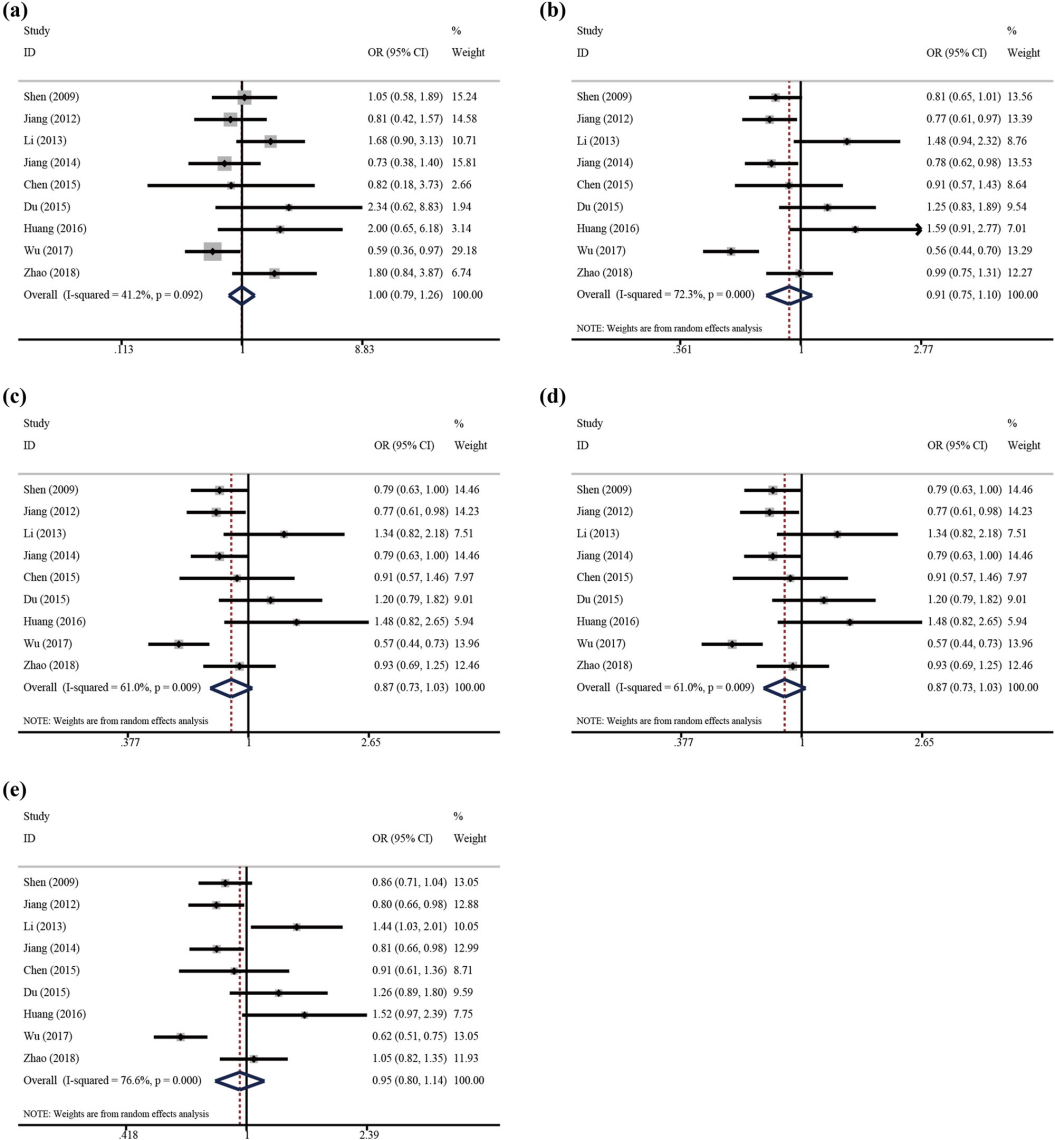
OR and 95% CIs of the associations between C-reactive protein (CRP) rs3093059 T/C polymorphism and IS risk: (a) CC vs TC + TT; (b) TT vs TC + CC; (c) TC vs TT; (d) CC vs TT; (e) C vs T.

**Figure 3:**
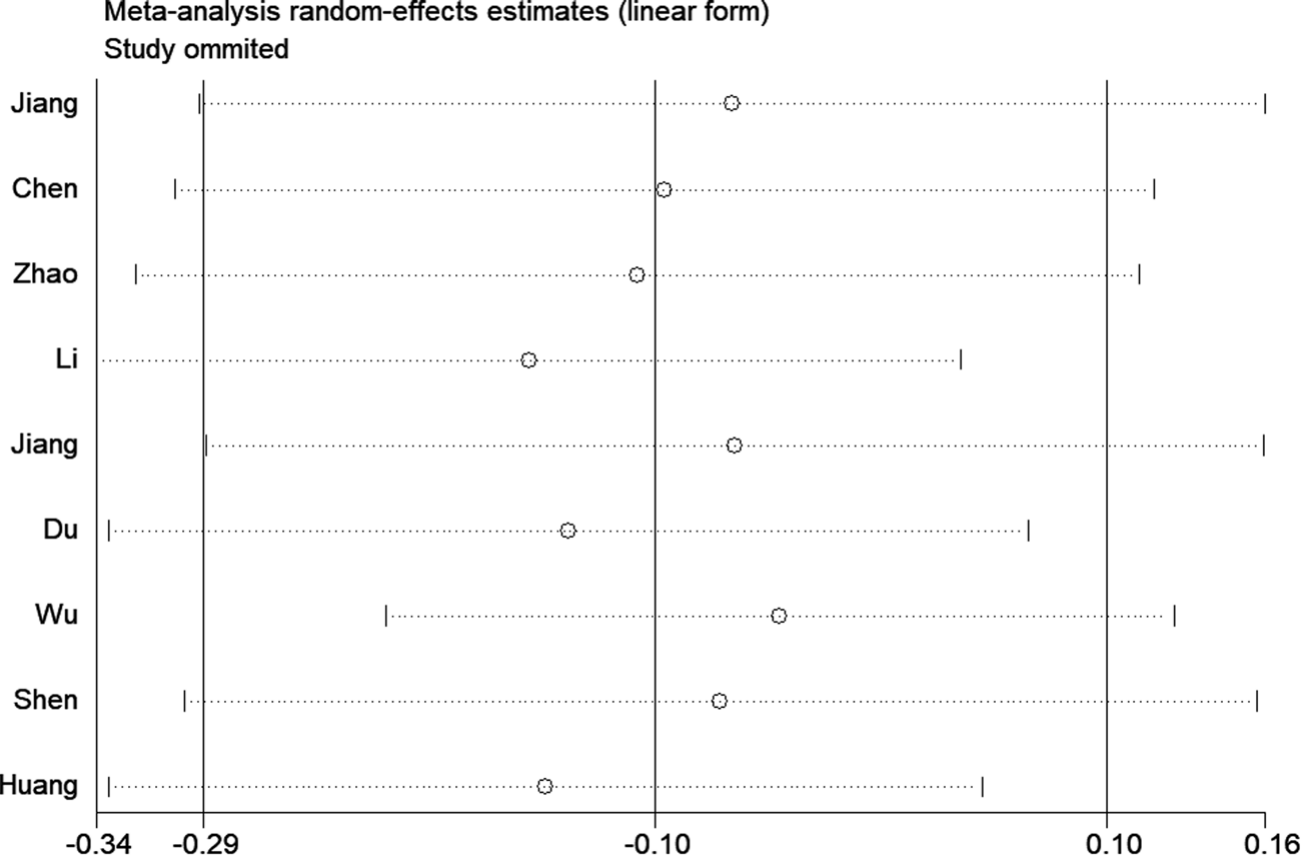
Sensitivity analysis was used to estimate the individual influence of studies on pooled results under the dominant genetic model.

The main results for the association between CRP rs3093059 T/C polymorphism and IS risk are summarized in Table 2. Based on global population, none of five genetic models indicated a significant association with IS risk (homozygote, CC vs TT: OR = 1.08, 95% CI 0.91–1.16, *p* = 0.637; recessive, CC vs TC + TT: OR = 1.00, 95% CI 0.79–1.26, *p* = 0.975; dominant, TT vs TC + CC: OR = 0.91, 95% CI 0.75–1.10, *p* = 0.327; homozygote, CC vs TT: OR = 1.06, 95% CI 0.71–1.58, *p* = 0.786; heterozygote, TC vs TT: OR = 0.87, 95% CI 0.73–1.03, *p* = 0.112; allele, C vs T: OR = 0.95, 95% CI 0.80–1.14, *p* = 0.592) (Figure 2). Moreover, the synthesized result suggested a null association between the rs3093059 T/C polymorphism and IS risk in the subgroup analysis according to source of controls, genotype method, and NOS score (Table 2). Significant between-study heterogeneities were observed in some genetic models; thus, to confirm the robustness of the meta-analysis, sensitivity analyses were necessary to be carried out. As shown in Figure 3, none of the studies affected the pooled result in the dominant genetic model, which suggested that our results were statistically robust. In addition, publication bias usually makes it difficult to have confidence in any reported differences. Thus, Begg’s and Egger’s linear regression tests were used to visualize publication bias, and the results of Begg’s test and Egger’s test suggested a statistically significant publication bias in heterozygous and allelic genetic models (Table 2). Therefore, we conducted the trim-and-fill method to make the OR value more reliable. It is interesting to note that OR value was significantly decreased (heterozygous genetic model: OR = 0.74; 95% CI 0.62–0.89, *p* = 0.001; allelic genetic model: OR = 0.78; 95% CI 0.64–0.95, *p* = 0.012). We used meta-regression to detect the influence covariates and found the source of controls (heterozygous genetic model: coefficient −0.349, 95% CI 0.500–0.994, *p*  =  0.047; allelic genetic model: coefficient −0.375, 95% CI 0.476–0.993, *p*  =  0.047) the influence factor.

**Table 2:** Overall and subgroup analyses for CRP rs3093059polymorphism and IS risk

Comparison	Studies	Overall effect			Heterogeneity		Publication bias	
		OR (95% CI)	*Z*-score	*p*-value	*I* ^2^ (%)	*p*-value	Begg’s test	Egger’s test
**Recessive genetic model**								
Overall	8	1.00 (0.79, 1.26)	0.03	0.975	41.2	0.092	0.404	0.165
PCR-RFLP	5	1.05 (0.78, 1.41)	0.33	0.741	8.5	0.362	—	—
Mass ARRAY	1	1.80 (0.84, 3.87)	1.50	0.133	—	—	—	—
TaqMan	2	0.73 (0.46, 1.14)	1.40	0.160	73.4	0.052		
HB	5	1.33 (0.95, 1.87)	1.67	0.094	16.1	0.310		
PB	3	0.76 (0.55, 1.06)	1.61	0.107	8.0	0.337		
NOS score <7	4	1.33 (0.90, 1.96)	1.44	0.150	0	0.522		
NOS score ≥7	4	0.85 (0.63, 1.14)	1.09	0.276	51.1	0.085		
**Dominant genetic model**								
Overall	8	0.91 (0.75, 1.10)	0.98	0.327	72.3	0.001	0.095	0.015
PCR-RFLP	5	0.91 (0.76, 1.09)	1.05	0.294	53.5	0.056		
Mass ARRAY	1	0.99 (0.75, 1.31)	0.07	0.947	—	—		
TaqMan	2	0.91 (0.33, 2.54)	0.18	0.856	91.4	0.001		
HB	5	1.07 (0.85, 1.34)	0.57	0.570	56.4	0.043		
PB	3	0.70 (0.56, 0.88)	3.01	0.003	66.2	0.052		
NOS score <7	4	1.04 (0.78, 1.40)	0.29	0.775	76.3	0.002		
NOS score ≥7	4	0.82 (0.64, 1.06)	1.49	0.136	59.9	0.058		
**Heterozygous genetic model**								
Overall	8	0.87 (0.73, 1.03)	1.59	0.112	61.0	0.009	0.037	0.013
PCR-RFLP	5	0.87 (0.75, 1.01)	1.78	0.075	31.2	0.201		
Mass ARRAY	1	0.93 (0.69, 1.25)	0.48	0.633	—	—		
TaqMan	2	0.88 (0.35, 2.24)	0.26	0.793	88.4	0.003		
HB	5	1.00 (0.83, 1.21)	0.01	0.997	34.4	0.179		
PB	3	0.71 (0.58, 0.87)	3.35	0.001	54.1	0.113		
NOS score <7	4	0.99 (0.76, 1.27)	0.11	0.911	43.6	0.150		
NOS score ≥7	4	0.80 (0.64, 1.01)	1.92	0.055	67.0	0.016		
Overall	8	1.06 (0.71, 1.58)	0.27	0.786	59.3	0.012	0.297	0.128
PCR-RFLP	5	1.04 (0.70, 1.55)	0.21	0.831	34.0	0.181		
Mass ARRAY	1	1.76 (0.82, 3.81)	1.45	0.148	—	—		
TaqMan	2	0.96 (0.21, 4.50)	0.05	0.962	83.5	0.014		
HB	5	1.43 (0.90, 2.28)	1.50	0.135	36.0	0.167		
PB	3	0.69 (0.44, 1.06)	1.69	0.092	40.8	0.185		
NOS score <7	4	1.36 (0.86, 2.14)	1.33	0.182	13.3	0.326		
NOS score ≥7	4	0.90 (0.52, 1.54)	0.40	0.690	65.3	0.021		
**Allelic genetic model**								
Overall	8	0.95 (0.80, 1.14)	0.54	0.592	76.6	0.001	0.037	0.015
PCR-RFLP	5	0.95 (0.80, 1.13)	0.56	0.579	63.8	0.017		
Mass ARRAY	1	1.05 (0.82, 1.35)	0.42	0.675	—	—		
TaqMan	2	0.95 (0.39, 2.28)	0.12	0.906	92.2	0.001		
HB	5	1.10 (0.89, 1.37)	0.89	0.373	65.4	0.013		
PB	3	0.75 (0.62, 0.92)	2.84	0.004	66.3	0.051		
NOS score <7	4	1.08 (0.82, 1.40)	0.54	0.590	66.1	0.031		
NOS score ≥7	4	0.87 (0.69, 1.10)	1.16	0.246	79.1	0.001		

OR, odds ratio; CI, confidence interval; HB, hospital-based source of control; PB, population-based source of control; PCR-RFLP, polymerase chain reaction-restriction fragment length polymorphism; NOS, Newcastle-Ottawa scale.

### 3.3 CRP rs1205 C/T polymorphism and IS risk

**Figure 4:**
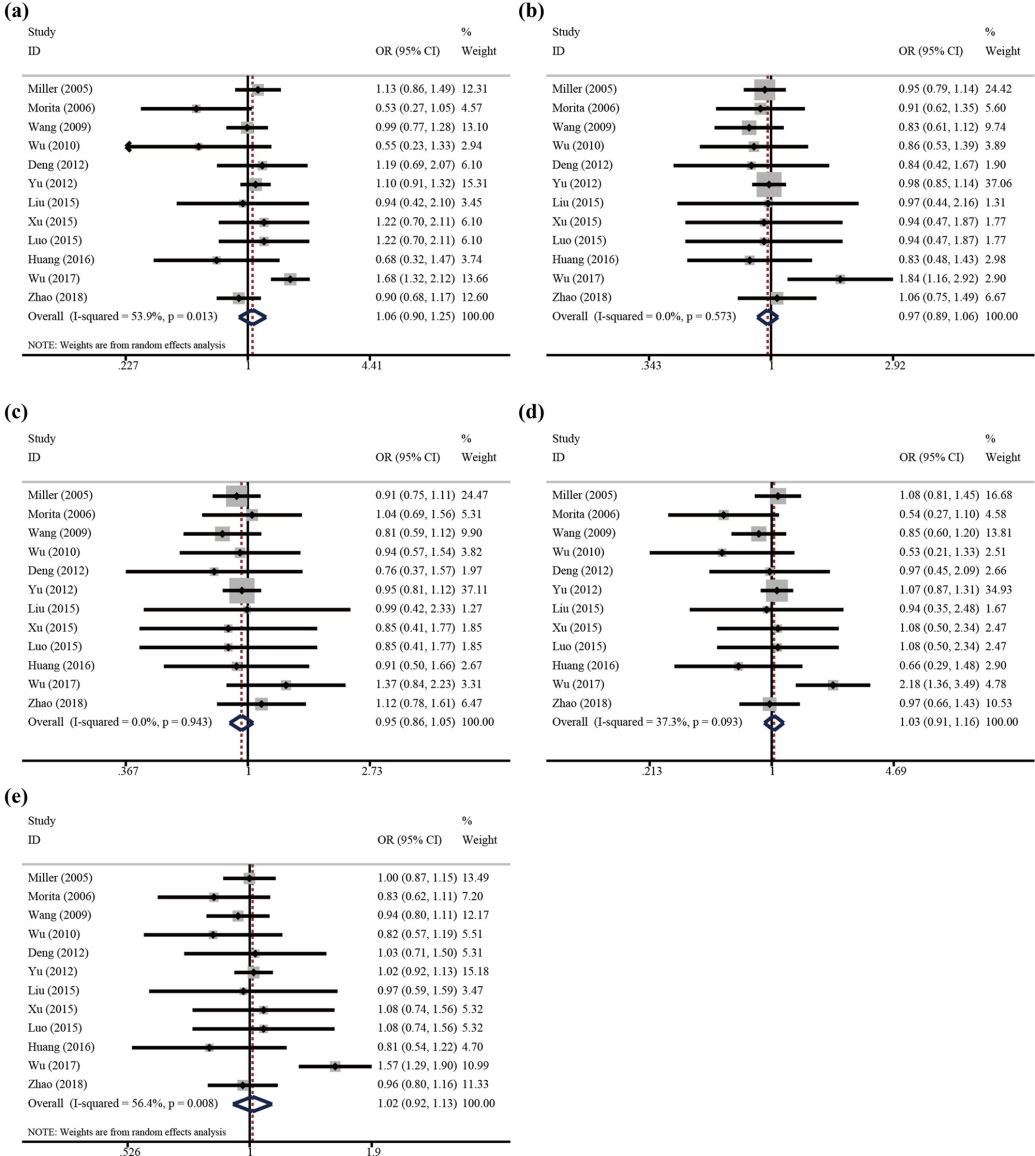
OR and 95% CIs of the associations between CRP rs1205 C/T polymorphism and IS risk: (a) TT vs CT + CC; (b) CC vs CT + TT; (c) CT vs CC; (d) TT vs CC; (e) T vs C.

**Figure 5:**
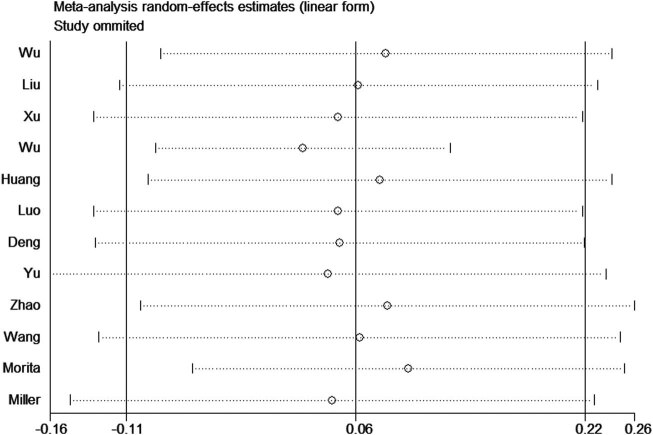
Sensitivity analysis was used to estimate the individual influence of studies on pooled results under the recessive genetic model.

The main results for the association between CRP rs1205 C/T polymorphism and IS risk are summarized in Table 3. The results indicated that there were no significant associations between CRP rs1205 C/T polymorphism and IS risk under homozygote (TT vs CC: OR = 1.08, 95% CI 0.91–1.16, *p* = 0.637), heterozygote (CT vs CC: OR = 0.91, 95% CI 0.75–1.11, *p* = 0.288), dominant (CC vs CT + TT: OR = 0.97, 95% CI 0.89–1.06, *p* = 0.524), recessive (TT vs CT + CC: OR = 1.06, 95% CI 0.90–1.25, *p* = 0.495), and allele (T vs C: OR = 1.02, 95% CI 0.92–1.13, *p* = 0.776 [Figure 4]). The synthesized result suggested a null association between the CRP rs1205 C/T polymorphism and IS risk in the subgroup analysis according to country, genotype method, source of controls, and NOS score (Table 3). Significant between-study heterogeneity was observed in some genetic models; thus, to confirm the robustness of the meta-analysis, sensitivity analyses were necessary to be carried out. As shown in Figure 5, none of the studies affected the pooled result, which suggested that our results were statistically robust. Begg’s and Egger’s linear regression tests were used to visualize publication bias, and the results of Begg’s test and Egger’s test suggested no statistically significant publication bias in all genetic models (Table 3).

**Table 3:** Overall and subgroup analyses for CRP rs1205 polymorphism and IS risk

Comparison	Studies	Overall effect			Heterogeneity		Publication bias	
		OR (95% CI)	*Z*-Score	*p*-Value	*I* ^2^ (%)	*p*-Value	Begg’s test	Egger’s test
**Recessive genetic model**								
Overall	12	1.06 (0.90, 1.25)	0.68	0.495	53.9	0.013	0.244	0.175
PCR-RFLP	6	1.09 (0.94, 1.28)	1.14	0.253	0	0.725	—	—
Mass ARRAY	1	0.90 (0.68, 1.17)	0.80	0.426	—	—	—	—
TaqMan	5	1.03 (0.74, 1.44)	0.19	0.850	77.3	0.001	—	—
HB	9	1.02 (0.91, 1.15)	0.34	0.731	0	0.690	—	—
PB	3	1.11 (0.68, 1.80)	0.40	0.687	83.4	0.002	—	—
NOS score <7	4	1.07 (0.93, 1.23)	0.89	0.376	0	0.866	—	—
NOS score ≥7	8	1.01 (0.77, 1.33)	0.08	0.935	69	0.002	—	—
China	10	1.09 (0.91, 1.30)	0.94	0.349	53.2	0.023	—	—
Japan	2	0.83 (0.40, 1.71)	0.50	0.615	75.5	0.043	—	—
**Dominant genetic model**								
Overall	12	0.97 (0.89, 1.06)	0.64	0.524	0	0.573	0.837	0.935
PCR-RFLP	6	0.96 (0.84, 1.10)	0.54	0.588	0	0.994	—	—
Mass ARRAY	1	1.06 (0.75, 1.49)	0.30	0.761	—	—	—	—
TaqMan	5	0.97 (0.84, 1.10)	0.51	0.610	54.8	0.065	—	—
HB	9	0.95 (0.85, 1.06)	0.95	0.341	0	0.984	—	—
PB	3	1.02 (0.87, 1.19)	0.24	0.811	71.9	0.028	—	—
NOS score <7	4	0.95 (0.84, 1.08)	0.77	0.444	0	0.794	—	—
NOS score ≥7	8	0.99 (0.87, 1.13)	0.14	0.889	16	0.304	—	—
China	10	0.98 (0.88, 1.10)	0.29	0.770	3.4	0.408	—	—
Japan	2	0.94 (0.80, 1.11)	0.72	0.471	0	0.863	—	—
**Heterozygous genetic model**								
Overall	12	0.91 (0.75, 1.11)	1.06	0.288	0	0.943	0.732	0.857
PCR-RFLP	6	0.94 (0.81, 1.08)	0.91	0.362	0	0.991	—	—
Mass ARRAY	1	1.12 (0.78, 1.61)	0.60	0.548	—	—	—	—
TaqMan	5	0.94 (0.81, 1.08)	0.88	0.380	0	0.496	—	—
HB	9	0.93 (0.83, 1.05)	1.11	0.265	0	0.974	—	—
PB	3	0.98 (0.83, 1.16)	0.27	0.790	15.2	0.308	—	—
NOS score <7	4	0.92 (0.80, 1.06)	1.16	0.248	0	0.837	—	—
NOS score ≥7	8	0.98 (0.85, 1.12)	0.35	0.726	0	0.828	—	—
China	10	0.96 (0.85, 1.07)	0.78	0.436	0	0.883	—	—
Japan	2	0.93 (0.78, 1.12)	0.75	0.453	0	0.587	—	—
**Homozygous genetic model**								
Overall	12	1.08 (0.91, 1.16)	0.47	0.637	37.3	0.093	0.244	0.380
PCR-RFLP	6	1.03 (0.86, 1.23)	0.33	0.739	0	0.823	—	—
Mass ARRAY	1	0.97 (0.66, 1.43)	0.13	0.894	—	—	—	—
TaqMan	5	1.04 (0.87, 1.26)	0.44	0.661	73.8	0.004	—	—
HB	9	0.97 (0.84, 1.13)	0.35	0.725	0	0.849	—	—
PB	3	1.19 (0.95, 1.49)	1.48	0.140	82.5	0.003	—	—
NOS score <7	4	1.01 (0.85, 1.19)	0.09	0.930	0	0.731	—	—
NOS score ≥7	8	1.06 (0.88, 1.26)	0.59	0.554	56.6	0.024	—	—
China	10	1.05 (0.91, 1.20)	0.66	0.512	36.7	0.115	—	—
Japan	2	0.97 (0.74, 1.27)	0.25	0.804	68.3	0.076	—	—
**Allelic genetic model**								
Overall	12	1.02 (0.92, 1.13)	0.28	0.776	56,4	0.008	0.945	0.665
PCR-RFLP	6	1.01 (0.93, 1.11)	0.29	0.772	0	0.916	—	—
Mass ARRAY	1	0.96 (0.80, 1.16)	0.39	0.699	—	—	—	—
TaqMan	5	1.02 (0.81, 1.28)	1.13	0.260	82.6	0.001	—	—
HB	9	0.99 (0.92, 1.06)	0.40	0.691	0	0.927	—	—
PB	3	1.10 (0.78, 1.56)	0.54	0.586	89.1	0.001	—	—
NOS score <7	4	1.00 (0.92, 1.09)	0.03	0.976	0	0.839	—	—
NOS score ≥7	8	1.01 (0.86, 1.20)	0.15	0.884	70.6	0.001	—	—
China	10	1.03 (0.91, 1.17)	0.52	0.601	60.7	0.006	—	—
Japan	2	0.95 (0.82, 1.12)	0.58	0.560	20.3	0.263	—	—

OR, odds ratio; CI, confidence interval; HB, hospital-based source of control; PB, population-based source of control; PCR-RFLP, polymerase chain reaction-restriction fragment length polymorphism; NOS, Newcastle-Ottawa scale.

## 4 Discussion

This meta-analysis included the literature published in recent years about the association between CRP polymorphisms (rs3093059, rs1205) and IS susceptibility. The pooled results revealed that CRP polymorphisms (rs3093059, rs1205) might not associate with IS risk.

CRP, a glycoprotein released by the liver, has been regarded as an essential mediator and a hallmark of the acute-phase response to inflammation and is recommended for use in risk assessment in IS patients [35]. CRP is an evolutionarily conserved protein with a unique pentameric structure and binds to ligands in a calcium-dependent manner [36]. Once binding to ligands, CRP interacts with the classical complement pathway and Fcγ receptors to participate in the activation of the innate immune system [37,38]. The gene of CRP is located on chromosome 1 and has only two exons and a single intron, including 204-amino acid peptides in the coding regions of exons and 280-base pairs in the domain of the intron [39]. Due to the existence of single-nucleotide polymorphisms (SNPs), CRP genetic variants and individual variations in the inflammatory response are significant. Many researchers have proved that the altered serum level of CRP should be attributed to the CRP gene variations on chromosomes 1q21 to 1q23 [40]. A couple of sequence variations at this locus have been shown to modulate plasma CRP levels and the risk of IS [41]. So the SNPs of CRP are a vital factor in the development of IS.

As an inflammation-associated protein, CRP can be divided into two structurally and functionally independent forms: (1) net anti-inflammatory, serum-associated native pentameric CRP, and (2) pro-inflammatory tissue-associated, monomeric CRP (mCRP) [37,42]. Several studies proved that a dramatic increase in the expression of mCRP had been observed in blood vessels of damaged brain regions in IS patients [43,44,45]. Krupinski and his colleagues found a higher expression of mCRP within microvessels with unstable plaques while normal-looking arteries, and stable fibrous lesions contained a significantly lower expression [44]. It suggested that mCRP may have a pathological role in developing unstable atherosclerosis and/or increased risk of plaque thrombosis, which could lead to the occurrence of IS. mCRP increases the activation of the inflammation both *in vitro* and *in vivo*, getting deposited chronically within the brain after IS, and may play a role in perpetuating neuroinflammation after brain injury [35]. Of course, there are still some opposite opinions. The function of CRP in the development of IS should be studied further.

Though the clear mechanism is ambiguous, CRP is closely related to the occurrence, development, and outcome of IS. The results of clinical studies show that CRP levels increase in the first 48 h after onset, are still elevated at 7 days and remain high for 3–6 months after IS [46,47]. CRP levels correlate with IS severity and can be a marker of IS etiology, with higher CRP in more severe cardioembolic or large artery disease stroke than in stroke caused by small artery disease [47,48,49]. Numbers of clinical studies use CRP as a biomarker to predict the occurrence of IS, and try to explore the relationship between SNPs of CRP and IS. The effect of CRP SNPs such as rs1800947, rs1417938, rs1130864, and rs3093077 on circulating protein level and the outcome has been assessed in a cohort of in-patients with cardiovascular diseases (e.g., IS) by Schulz et al. They found that both CRP level ≥5 mg/L and SNP rs1800947 of the CRP gene were independent risk factors for further adverse vascular events among patients with cardiovascular diseases within a 3-year follow-up [50]. A study of clinical samples by Williams et al. found that SNPs at rs3093068, rs16842599, and rs11265260 loci of CRP were associated with the occurrence and recurrence of IS [51]. Manuela and his colleagues consider that CRP levels after a minor first cerebrovascular event (transient ischemic attack or lacunar stroke) can contribute to identifying patients at high risk of a second ischemic event. Rs3093059 is located in the promoter region of the CRP gene. The mutation of this site would provide convenience for LHX2 binding to promote expression [52]. C alleles at rs3093059 were positively associated with increased CRP elevation in IS patients, which is inconsonant with our results. After multivariate adjustment, rs3093059 was found to be associated with decreased IS risk in the Chinese population [24]. Also, no association was detected between CRP gene polymorphisms and IS risk in the Swedish [41] population and Indian population [53]. It suggested that whether Rs3093059 can be judged as a risk factor in IS cases may be related to the population and environment. The rs1205 was located in the untranslated region of the CRP gene region. It was reported that CRP rs1205 polymorphism is associated with elevated CRP levels in Aortic stenosis patients and cardioembolic stroke [54]. However, it was found a negative association in our study is attributed to the type of stroke and the underlying condition of the patient. Circulating levels of CRP could be influenced by age, obesity, sex, smoking, diabetes, and use of medications summarily [55].

To our knowledge, this study is the first meta-analysis to focus on CRP polymorphisms (rs3093059 T/C and rs1205 C/T) and IS risk and proved CRP rs3093059 T/C and rs1205 C/T polymorphisms have little association with the risk of IS. Based on the aforementioned analysis, our study still has some limitations and shortages. On the one hand, the data are still relatively small and may not provide sufficient power to estimate the association between CRP gene polymorphisms and IS risk. Few studies have investigated the association between the CRP gene and patients’ stroke subtypes and patient-based characteristics, which has to be confirmed in more populations. On the other hand, as a type of retrospective study, a meta-analysis may encounter recall or selection bias, possibly influencing the reliability of our study results. Therefore, more studies with larger sample sizes are needed to accurately provide a more representative conclusion.

## 5 Conclusion

The current meta-analysis result suggests that CRP (rs3093059 T/C and rs1205 C/T) polymorphisms might not be associated with the risk of IS. In addition, CRP genetic variant might be associated with multiple internal and external factors, which suggests that further efforts are needed to dissect subgroups and patients’ overall physical condition. However, large sample size and well-designed studies within different ethnic are needed to confirm the findings of our study
